# Prospects for daily online adaptive radiotherapy via ethos for prostate cancer patients without nodal involvement using unedited CBCT auto‐segmentation

**DOI:** 10.1002/acm2.13399

**Published:** 2021-08-25

**Authors:** Mojtaba Moazzezi, Brent Rose, Kelly Kisling, Kevin L. Moore, Xenia Ray

**Affiliations:** ^1^ Department of Radiation Medicine and Applied Sciences University of California San Diego La Jolla California USA

**Keywords:** Adaptive Radiation Therapy, Auto‐planning, Auto‐segmentation, Cone‐Beam CT, Online Adaptation, Prostate Cancer

## Abstract

**Purpose:**

Implementing new online adaptive radiation therapy technologies is challenging because extra clinical resources are required particularly expert contour review. Here, we provide the first assessment of Varian's Ethos™ adaptive platform for prostate cancer using no manual edits after auto‐segmentation to minimize this impact on clinical efficiency.

**Methods:**

Twenty‐five prostate patients previously treated at our clinic were re‐planned using an Ethos™ emulator. Clinical target volumes (CTV) included intact prostate and proximal seminal vesicles. The following clinical margins were used: 3 mm posterior, 5 mm left/right/anterior, and 7 mm superior/inferior. Adapted plans were calculated for 10 fractions per patient using Ethos's auto‐segmentation and auto‐planning workflow without manual contouring edits. Doses and auto‐segmented structures were exported to our clinical treatment planning system where contours were modified as needed for all 250 CTVs and organs‐at‐risk. Dose metrics from adapted plans were compared to unadapted plans to evaluate CTV and OAR dose changes.

**Results:**

Overall 96% of fractions required auto‐segmentation edits, although corrections were generally minor (<10% of the volume for 70% of CTVs, 88% of bladders, and 90% of rectums). However, for one patient the auto‐segmented CTV failed to include the superior portion of prostate that extended into the bladder at all 10 fractions resulting in under‐contouring of the CTV by 31.3% ± 6.7%. For the 24 patients with minor auto‐segmentation corrections, adaptation improved CTV D98% by 2.9% ± 5.3%. For non‐adapted fractions where bladder or rectum V90% exceeded clinical thresholds, adaptation reduced them by 13.1% ± 1.0% and 6.5% ± 7.3%, respectively.

**Conclusion:**

For most patients, Ethos's online adaptive radiation therapy workflow improved CTV D98% and reduced normal tissue dose when structures would otherwise exceed clinical thresholds, even without time‐consuming manual edits. However, for one in 25 patients, large contour edits were required and thus scrutiny of the daily auto‐segmentation is necessary and not all patients will be good candidates for adaptation.

## 1 INTRODUCTION

For patients with prostate cancer, the position of the clinical target volume (CTV) can move up to 1.5‐2.2 cm between treatment fractions due to physiologic changes in the bladder and rectum volume.^1^ While online image guidance can help overcome the translational portions of this variation,^2^ the prostate and seminal vesicles have been shown to move independently^3^ of each other that can result in compromised alignment at treatment. This in turn may lead to either decreased dose to the CTV risking loss of tumor control,^4^ or increased dose to normal tissues that can move in‐field.^3^

Several groups have investigated different adaptive radiation therapy techniques for prostate to overcome these challenges. Park et al acquired full computed tomography (CT) scans for the first 5 days of treatment for almost 1000 patients to design patient‐specific margins for motion that resulted in an average decrease in the planning target volume (PTV) of 74.4 ± 30.2 cc and excellent patient outcomes.^5^ However, this approach required substantial clinical resources (time on the simulation CT, fusion of the extra images, and manual re‐planning prior to the second week of treatment). Mestrovic et al demonstrated that intra‐fractional digital tomosynthesis images could be used to adapt each subsequent portion of the field in a treatment plan for a virtual phantom.^6^ But it is unclear if this approach could work in patients because the added heterogeneity may make identifying tissue boundaries challenging when images are reconstructed from only a subset of the X‐ray projections. A new approach using kV images along with multi‐leaf collimator tracking^7^ to track and adapt the plan in real‐time was recently used prospectively in the SPARK trial for 48 prostate patients.^8^ This method is clinically efficient and can account for both inter‐ and intra‐fraction motion. However, it also relies on the invasive placement of fiducials or electromagnetic transponders for prostate monitoring. Additionally, it cannot visualize the seminal vesicles or organs‐at‐risk (OAR) and thus cannot optimize the plan for daily changes in their positions.

Ethos™ (Varian Medical Systems, Palo Alto, CA) is a novel commercial linear accelerator that includes an online adaptive radiation therapy workflow based on high quality iterative cone‐beam CT (iCBCT) images.^9^ In comparison to the technologies described above Ethos (a) auto‐segments both the target and organs‐at‐risk on the daily images and (b) optimizes an entirely new plan to balance tumor coverage and normal tissue sparing. However, implementation of any adaptive workflow comes with a cost to clinical efficiency^10^ because adaptation requires extra computational time to auto‐segment/auto‐plan and will require extra person‐hours since the output of the auto‐segmentation needs expert review.^11^ This last component can be the most time‐consuming and may be unnecessary as long as the errors in the auto‐segmentation are small and the clinical CTV‐to‐PTV margins are large enough to account for minor errors. This possibility must be carefully investigated using retrospective patient data to inform prospective implementation of this new technology.

The purpose of this study was to evaluate if a dosimetric benefit exists for a daily online adaptive radiation therapy workflow for prostate based on cone‐beam CT images in which the Ethos auto segmentation results were accepted without modifications. Thus, this approach allows us to evaluate whether the delivered dose to the tumor can be improved even when the time allowed for adaptation is minimized.

## 2 METHODS

**FIGURE 1:**
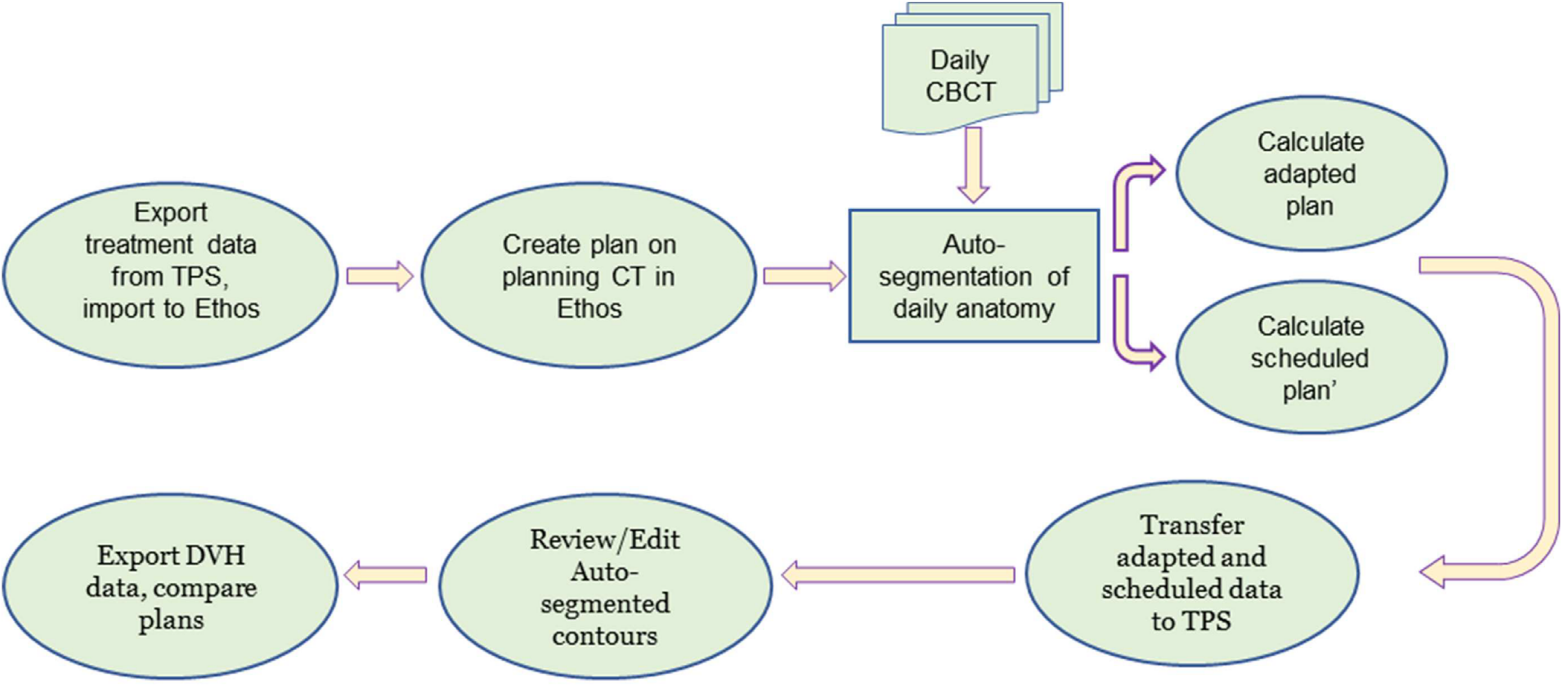
A schematic overview of our workflow for evaluating prostate adaptive radiotherapy via Ethos

Planning and on‐treatment imaging data from 25 intermediate‐risk prostate cancer patients without nodal involvement previously treated on our clinic's Varian Halcyon™ (Varian Medical Systems, Palo Alto, CA) were studied (UCSD IRB #200135). These patients had been aligned daily with iCBCT on the Halcyon, which utilizes identical iCBCT imaging equipment as the Ethos machine. The clinical plans delivered 54 Gy in 2 Gy fractions to the prostate and seminal vesicles and then a sequential boost of 24 Gy in 2 Gy fractions to just the prostate. The same clinically approved planning CT and structure set were used to generate the 54 Gy plans on a Varian Ethos™ emulator (Varian Medical Systems, Palo Alto, CA) while the boost plans were not evaluated in this study. The emulator software is identical to the commercially available Ethos treatment planning system MR1. Then the adaptation process was simulated for the first 10 fractions for each patient using their previous on‐treatment CBCTs and the Ethos emulator. This process includes auto‐segmentation of daily anatomy, calculation of the scheduled dose (dose from the non‐adapted plan on the daily anatomy), and optimization and calculation of the adapted dose. The auto‐segmentation, scheduled dose, and adapted dose for each fraction were exported to our clinical treatment planning system (TPS) for subsequent segmentation corrections and extraction of dose metrics to assess CTV dose and OAR sparing. In Figure 1, a graphical overview of the workflow for this study is provided. Specific details for each of these steps are described below.

### 2.1 Ethos emulator and adaptive workflow

Ethos includes a novel treatment planning system as well as on‐treatment auto‐segmentation and auto‐planning software. The original treatment plan and the daily adapted plans are created via a new artificial intelligence based Intelligent Optimization Engine™ (IOE).^12^ The IOE translates a set of prioritized clinical goals provided by the user into optimization objectives for the photon optimizer. It will also generate a ring structure and crop the OARs from the PTV for optimization. During optimization, the IOE adjusts the objective priorities to maximize an overall plan quality metric value. A RapidPlan™ model, which is a knowledge‐based planning tool,^13^, ^14^, ^15^, ^16^ can optionally be provided to the IOE to generate estimates of the DVH lower bounds that are then used as line objectives during optimization. When the optimization completes, the user is presented with a preview of the dose distribution and dose‐volume histograms (DVH) for each structure that they can adjust by re‐ordering their prioritized list of clinical goals. Approving the dose preview will generate a treatable plan that can then at each fraction be delivered as‐is (the scheduled plan) or re‐optimized to the anatomy‐of‐the‐day (the adapted plan).

For the adaptive workflow, Ethos first auto‐segments the daily CBCT images in three steps^12^: (a) auto‐segment the influencer structures (tissues whose boundaries affect the generated target's position; for prostate plans these are the prostate, seminal vesicles, rectum, bladder, and bowel) via an artificial intelligence algorithm based on a convolutional neural network, (b) auto‐segment the targets via a structure‐guided deformable image registration,^17^ and (c) auto‐segment the non‐influencer OAR structures by deformably registering the planning image to the session image. During the online adaptive workflow, the user may manually edit the contours after either step 1 or 2. Once the CTV has been approved, the predefined CTV‐to‐PTV margin is applied to create the target.

The dose from the scheduled and adapted plans are calculated on a simulated CT. Ethos generates this simulated CT by deformably registering the planning CT to the daily CBCT using the commercial B‐spline deformation model, Velocity™.^18^ To calculate the scheduled dose, the planning CT is rigidly registered to the CBCT, then the plan is recalculated on the simulated CT. The rigid registration uses three degrees of freedom because the Ethos couch cannot rotate. The process is automated and is initialized by matching the treatment isocenter from the planning CT to the imaging isocenter of the CBCT and then works to match the target volumes. No manual adjustments to the registration are possible. To calculate the adaptive dose, the IOE reoptimizes the plan on the simulated CT with the auto‐segmented anatomy from the CBCT using the same prioritized clinical goals that were used to create the initial plan.

In this study, we utilized an Ethos emulator to retrospectively generate the auto‐segmented anatomy‐of‐the‐day. The emulator was then used to calculate the scheduled and adapted doses for each treatment fraction as if we were treating in real‐time.

### 2.2 Auto‐planning

Ethos plans were created for the 25 prostate patients in this study using their clinical structure set and our standard asymmetric CTV‐to‐PTV margins: 3 mm posterior, 5 mm left/right/anterior, and 7 mm superior/inferior. The CTV included the intact prostate and proximal seminal vesicles. New plans were calculated using 12‐field IMRT and a prescription dose of 54 Gy in 2 Gy fractions to the generated PTV. For the planning goals used by the Ethos IOE, we used our institutional standards and guidelines scaled for the 54 Gy prescription dose, as shown in Table 1. These same DVH criteria were used to evaluate the daily adapted plans for improvements in OAR sparing, while D98% was used to assess changes in the CTV dose. Our clinically validated knowledge‐based planning model for prostate^19^, ^20^, ^21^ was also included in the plan optimization. Final plans were normalized so that 95% of the PTV was covered by the 100% isodose line.

**TABLE 1:** Institutional guidelines and ethos template goals for prostate plans

Ethos assigned priority		Structure	Institutionalclinical values (54 Gy + 24 Gy boost)	Ethos planning goals (54 Gy plan only)	Reportedmetrics for this study
1	Most Important	PTV	D95.0% ≥100.0%	D95.0% ≥100%	–
2	Very Important	CTV	D_mean_ ≥100.0%	D_mean_ ≥100%	D98%
	Very Important	PTV	D_max_ < 107.0%	D_max_ (0.10 cm^3^) < 107.0%	–
	Very Important	PTV	D_min_ > 95.0%	D_min_ (0.10 cm^3^) > 95.0%	–
	Very Important	Rectum	V70Gy < 20.0%	V48.50 Gy < 20.0%	V90% < 20.0%
	Very Important	Rectum	V60Gy < 25.0%	V41.50 Gy < 25.0%	V75 % < 25.0%
	Very Important	Rectum	V40Gy < 35.0%	V27.70 Gy < 35.0%	V50% < 35.0%
3	Important	Bladder	V70Gy < 25.0%	V48.50 Gy < 25.0%	V90% < 25.0%
	Important	Bladder	V60Gy < 35.0%	V41.50 Gy < 35.0%	V75 % < 35.0%
	Important	Bladder	V40Gy < 45.0%	V27.70 Gy < 45.0%	V50 % < 45.0%
	Important	Bowel	D_max _< 54.0 Gy	D_max_ < 27.40 Gy	–
4	Less Important	Femur left	V50Gy < 10.0%	V34.60 Gy < 10.0%	–
	Less Important	Femur right	V50Gy < 10.0%	V34.60 Gy < 10.0%	–
	Less Important	Penile bulb	D_mean _< 52.5 Gy	D_mean_ < 26.06 Gy	–

### 2.3 Auto‐segmentation

Each of the 25 prostate plans was adapted for the first 10 fractions using the Ethos Emulator. For adaptive planning to best fit a tight clinical workflow, the auto‐segmentation should be robust and require minimal or ideally no adjustments during treatment. To evaluate this potential approach, we did not edit the auto‐segmented contours during the simulated‐adaptive treatments. Instead, the auto‐segmented contours by Ethos were approved and used to create the adapted plans. Then these auto‐segmented contours, the calculated dose from the adapted plan, and the calculated dose from the scheduled plan on each day's CT were exported to our clinical treatment planning system (Eclipse™ version 15.6). Then to establish ground truth contours, all segmentations for CTV, bladder, and rectum were reviewed/edited by a medical physicist, and subsequently all CTVs were reviewed/edited by a physician with a clinical focus in prostate cancer. The auto‐segmentation was evaluated by comparing DVH metrics from auto‐segmented versus corrected contours. Dosimetric improvements were measured by comparing adapted and scheduled DVH metrics from the corrected contours.

### 2.4 Data extraction and analysis

The DVH data for the scheduled and adapted doses on the auto‐segmented and corrected contours were exported in batch from our TPS as JavaScript Object Notation (JSON) files using a stand‐alone executable script in C# using the functionality of ClearCheck™ (Radformation, Inc, New York, NY). MATLAB (MathWorks, Natick, MA) was used to parse the JSON files and analyze the data.

For the CTV, we calculated the percent difference in the volume between CTV_true_ and CTV_auto_ and qualitatively assessed where changes to the auto‐segmentation were most frequently made. To evaluate the impact of the errors in the auto‐segmentation, we analyzed the change in CTV DVH metrics. Specifically, we calculated the percent of fractions where the CTV_true_ was covered up to 98% of its volume (CTV D98%) by relative isodose lines (0‐100%) with and without adaptation. We also calculated the percent of fractions where the CTV_auto_ D98% was covered by each isodose line (0‐100%). The CTV_auto_ values act as a surrogate for the dose to the tumor that would be achieved if the CTV contours were edited prior to adaptation. The inter‐patient variability was assessed by comparing the median CTV_true_ D98% values from the scheduled and adapted plans for each patient.

To evaluate the rectum and bladder auto‐segmentation, changes in three DVH metrics (V90%, V75%, and V50% measured from the corrected versus auto‐segmented contours) were compared. To assess improvements in normal tissue sparing, these DVH metrics from the bladder and rectum were compared for the scheduled and adapted plans on the corrected contours. We also compared these values to our clinical goals to assess how often scheduled and adapted plans would have exceeded our thresholds. Finally, to evaluate if decreased dose to one OAR came at the expense of the CTV or the other OAR, we plotted the changes in each metric for the bladder versus the changes in the rectum and CTV metrics.

## 3 RESULTS

### 3.1 Auto‐segmentation

**FIGURE 2:**
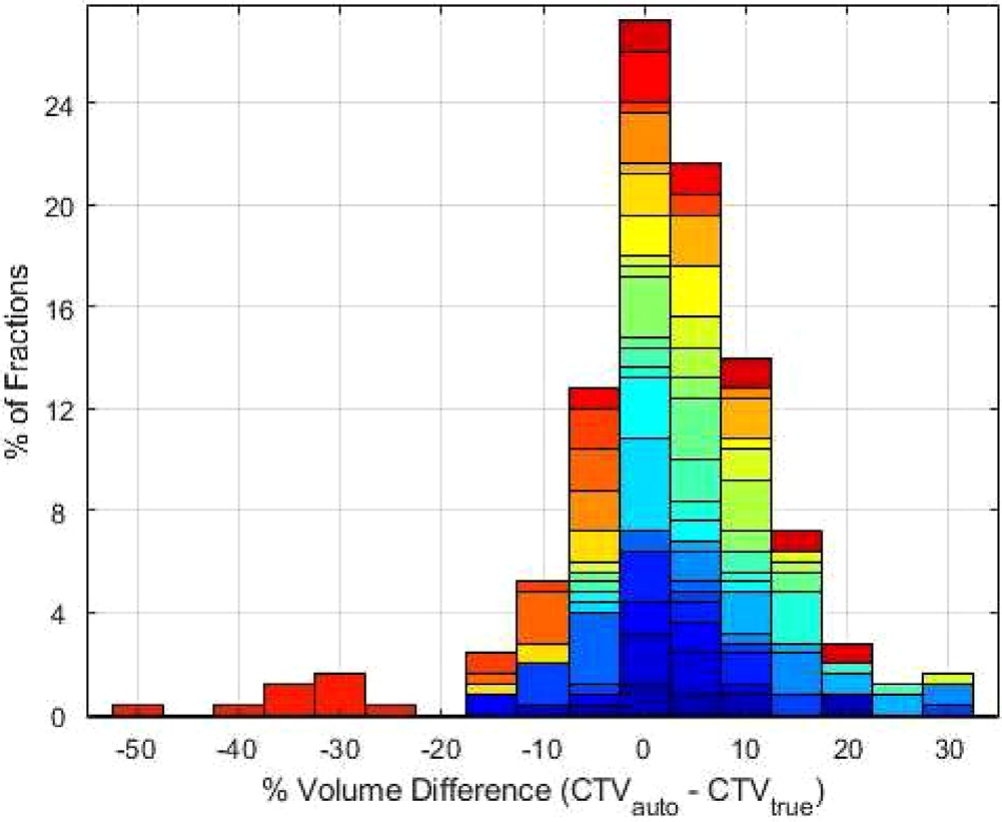
Volume differences (%) between the auto‐segmented CTV_auto_ and the corrected CTV_true_ for 25 patients (10 fractions each). Each patient is represented in a different color. While almost all CTV_auto_ contours required some editing, 70% of these edits were less than 10% of the CTV total volumes, and the average change in the volume difference was equal to 4.5%

**FIGURE 3:**
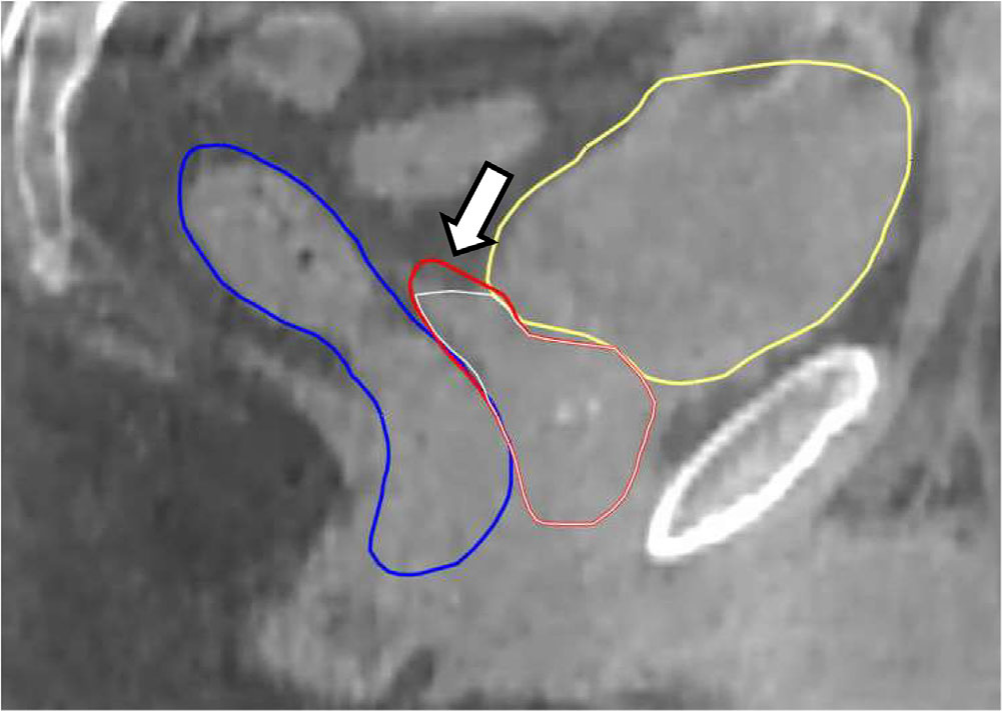
Examples of Ethos auto‐segmentation results. The auto‐segmented CTV (red), rectum (blue), and bladder (yellow) were generally auto‐segmented accurately though in some cases as shown the portion of the CTV corresponding to the proximal seminal vesicles extended further than our clinical guidelines of 1 cm. The corrected CTVtrue is shown in white

Overall 96% of fractions required auto‐segmentation edits, although corrections were generally minor (<10% of the volume for 70% of CTVs, 88% of bladders, and 90% of rectums). In 74% of all 250 fractions, the CTV_auto_ volume was larger than CTV_true_. Figure 2 shows the volume differences in CTV_auto_ and CTV_true_ for all patients. Contour edits were most often required to decrease the portion of seminal vesicles included within the CTV, while the intact prostate was generally auto‐segmented accurately (Figure 3).

**FIGURE 4:**
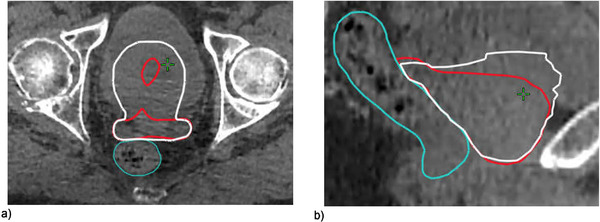
The auto‐segmentation only grossly under‐contoured a single outlier patient as shown in the (a) axial and (b) sagittal views above where the auto‐segmented CTV is in red and the corrected CTV is in white. For this case, edits were required to include superior slices of prostate that overlapped with bladder and to crop the SV resulting in overall volume increases of 25‐50%

There was one outlier patient with large volume changes of ‐25% to ‐50% (shown in Figure 2), where the Ethos auto‐segmentation had failed to include the superior portion of the prostate gland where it overlapped with bladder at each fraction. This error was visible on the pretreatment imaging (Figure 4) and would necessitate contour edits or using the scheduled plan to ensure that the CTV received appropriate dose.

**FIGURE 5:**
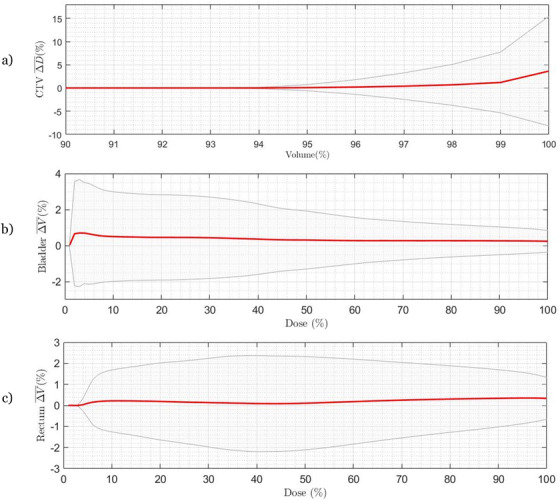
The average differences in the DVHs calculated from the auto‐segmented contour minus the corrected contour are plotted for 240 adapted plans (24 patients, 10 fractions each) for the (a) CTV, (b) bladder, and (c) rectum. Standard deviations are shown in grey. Overall, correcting the contours resulted in very minor differences of less than 1% to the DVH except for CTV D98% and higher where differences increased to 3.9% on average and up to 12.7%

Editing the contours for all the patients with the exception of the outlier resulted in small average changes in the adapted plan dose metrics: 0.7% ± 4.5% for CTV‐D98%, 0.3% ± 0.8% for Bladder‐V90%, and 0.3% ± 1.5% for Rectum‐V90%. In Figure 5, the average changes in the full DVH for all three structures are plotted to show these changes were consistently small at all DVH points.

### 3.2 Target dose

**FIGURE 6:**
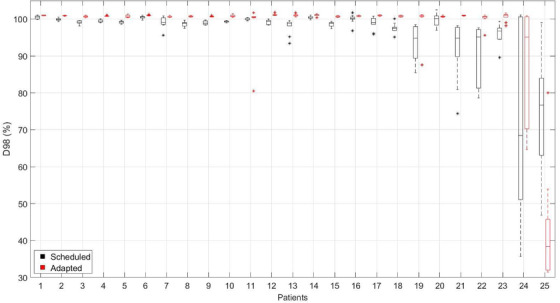
CTV_true_ D98% values for the 10 fractions per patient using the scheduled (black) versus adapted plans (red). Adaptation improved the CTV D98% values for all patients except one (patient 25) where the prostate auto‐segmentation had gross errors due to bladder overlap

The values for CTV_true_ D98% were plotted for each patient's 10 scheduled and adapted plans (Figure 6). For 24 patients, adaptation increased CTV_true_ D98% by 2.9% ± 5.3% on average. The outlier (patient 25) was the same patient with gross auto‐segmentation errors identified in the previous section who as a result experienced decreased values for CTV_true_ D98% with adaptation. Patients 19, 21, 22, and 23 had at least one fraction of their scheduled plan where the D98% < 90% and thus relatively larger improvements with adaptation compared to the rest of the cohort. Patient 24 also had a large improvement in the median value of D98% from 68.4% to 95.1% with adaptation, but for this patient, the seminal vesicles part of CTV_true_ had to be extended at some fractions, and thus CTV_true_ D98% had large variability ranging from 64.6% to 100.9%.

**FIGURE 7:**
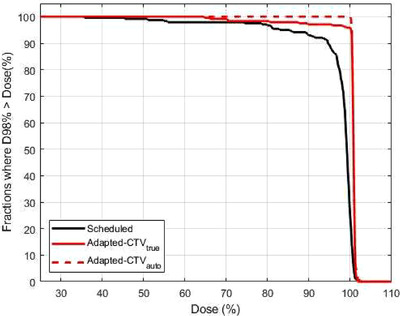
Fraction of treatments where CTV D98% was greater than a certain dose (x‐axis) with/without adaptation and/or time‐consuming manual CTV edits. Daily adaptation without manual contour edits at the machine (red solid line) improved CTV_true_ D98% compared to no adaptation (solid black line). In the adapted plans, the D98% measured from CTV_auto_ (red dashed line) was always greater than 99%. Thus editing the contours prior to plan adaptation at each fraction would likely achieve the same dose coverages for CTV_true_

To assess the overall rate of tumor coverage by different isodose lines, we calculated the percent of fractions where 98% of the CTV_true_ volume was covered by different relative dose levels for the adapted and scheduled plans, as shown in Figure 7. The outlier patient, (patient 25 from Figure 6) was excluded from this calculation as well as for the subsequent OAR analysis. The percent of fractions where CTV_auto_ was covered by each relative dose is also shown and acts as a surrogate for the CTV values if contour edits were performed prior to adaptation. Without adaptation, CTV_true_ D98% was greater than 95% for only 89% of fractions. Fully automated adaptation increased this value to 97% of fractions. Evaluating CTV D98% on CTV_auto_ increased this number to 100%, suggesting that if contours are edited on the Ethos platform prior to adaptation, the reoptimized adapted plan will always result in CTV D98% > 100%.

### 3.3 Normal tissue sparing

**FIGURE 8:**
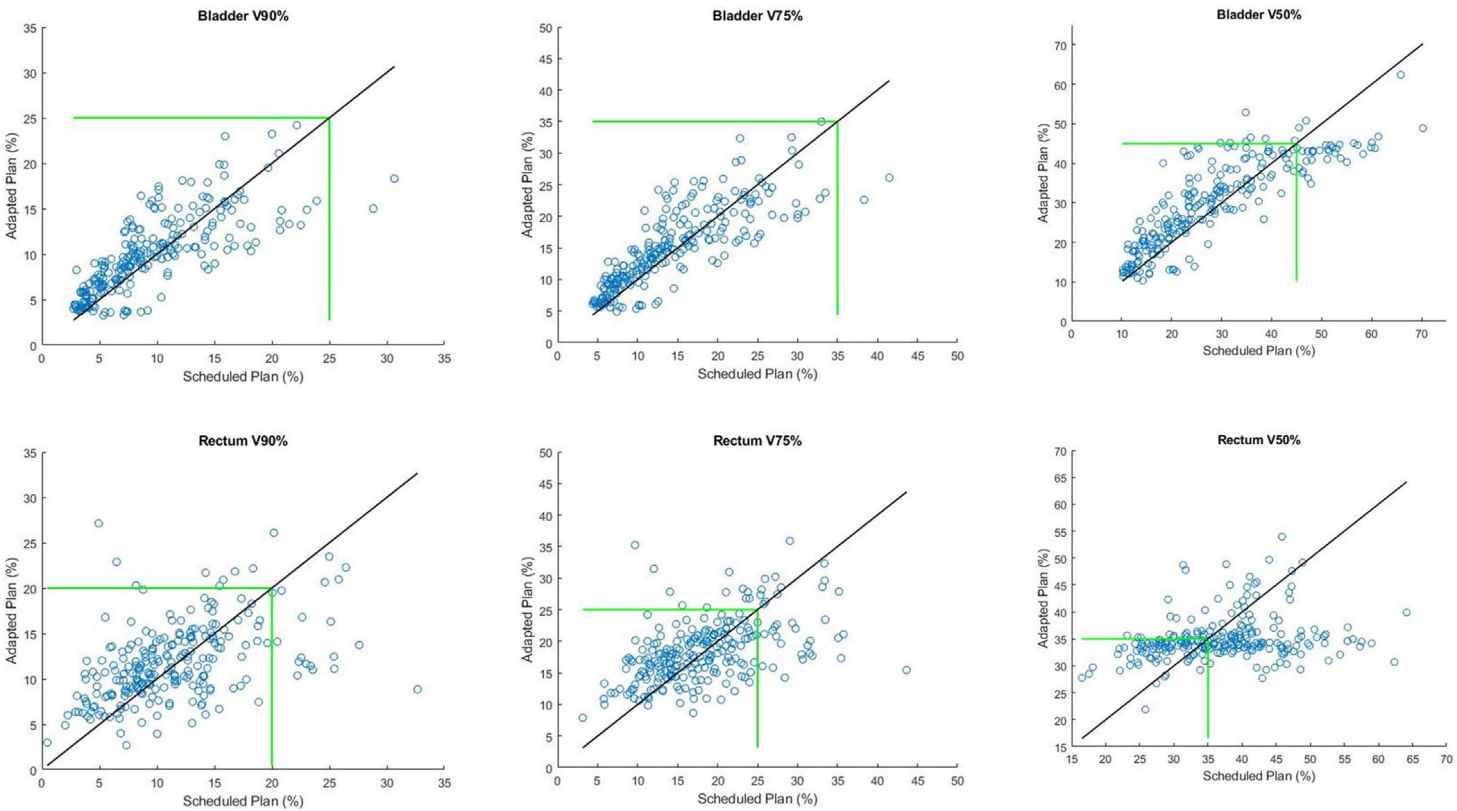
Comparison of bladder and rectum metrics for the adapted versus scheduled plans. Each point represents one fraction. The green line indicates our clinical threshold for each metric. Adaptation showed systematic improvement of metrics when the scheduled plans had values above the clinical threshold

The bladder and rectum DVH metrics are plotted for the adapted versus scheduled plans in Figure 8. All metrics were calculated from the corrected contours. For the bladder, the majority of the adapted plans (all V90% and V75% and 95% of V50%) and scheduled plans (99% of V90% and V75% and 85% of V50%) kept the three metrics within clinical thresholds (green line) and little systematic improvement was seen with adaptation for these fractions. However, for all fractions where the scheduled plan would have resulted in a value above the clinical threshold for Bladder V90% or V75%, the adapted plan lowered it to within tolerance. For Bladder V50% a systematic decrease was also seen with adaptation but there were also six fractions where adapting pushed the value beyond the clinical threshold and two fractions where the scheduled dose was beyond the clinical limit and adaptation further increased it. For the rectum, a similar trend was observed where adaptation did not systematically improve the metric values unless the values from the scheduled plan were above our tolerance value. This effect was most noticeable for Rectum V50%.

In Table 2, we calculated the average change in each metric with adaptation for plans with scheduled values above and below our clinical threshold. Above the threshold, large dose sparing was observed (6‐13% reduction in metric values with adaptation) while below the threshold, there was a slight increase in each metric (0.9‐3.2%).

**TABLE 2:** The average change in bladder and rectum metrics depended on whether the metric could meet the clinical goal in the scheduled plan

**Total number of fractions: 240**	Clinical goal(%)	# of fractionsabove limit	Number of fractionsbelow limit	Average change in metrics for fractions above the limit (%)	Average change in metrics for fractions below the limit (%)
Bladder V90%	<25	2	238	‐13.1 ± 1.0	1.0 ± 0.6
Bladder V75%	<35	2	238	‐6.7 ± 6.2	0.9 ± 3.9
Bladder V50%	<45	36	204	‐8.7 ± 5.4	3.1 ± 5.3
Rectum V90%	<20	20	220	‐6.5 ± 7.3	1.1 ± 4.0
Rectum V75%	<25	35	205	‐6.2 ± 7.6	1.3 ± 4.6
Rectum V50%	<35	136	104	‐7.0 ± 7.5	3.2 ± 4.0

**FIGURE 9:**
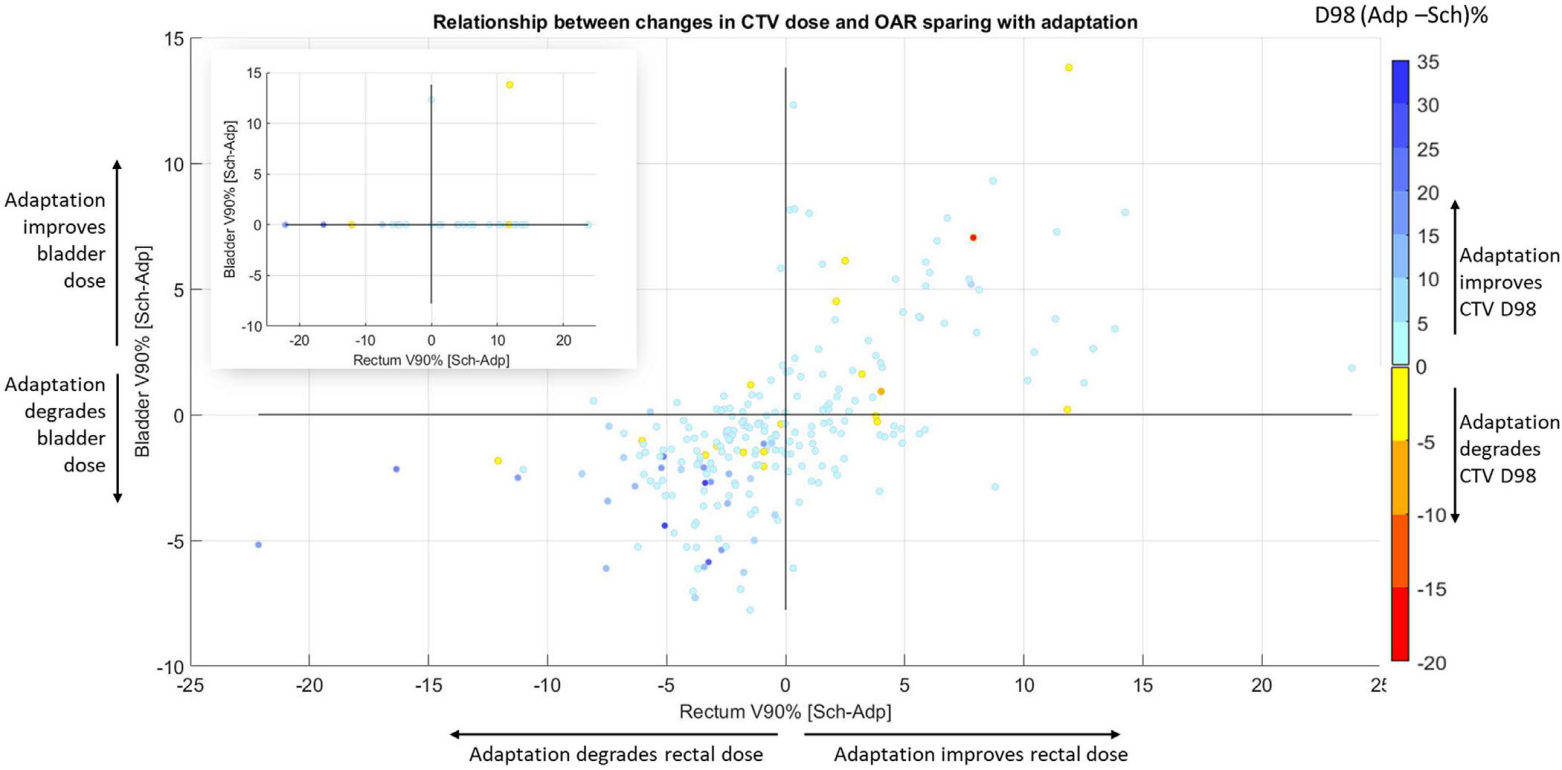
Relationship between changes in CTV dose and OAR sparing with adaptation. The relationship between changes with adaptation in Bladder V90% versus Rectum V90% is plotted while the change in CTV D98% is shown via color intensity. The in‐set plot in the top left, excluded fractions where changes in both Bladder V90% and Rectum V90% were within the clinical threshold. Additionally, for the inset plot if one of the bladder or rectum metrics was within the clinical threshold, the change in that metric is represented as 0 and falls on the x or y axis, respectively. Adp, adapted plan; Sch, Scheduled plan

Since target dose and OAR sparing are negatively correlated, the observed increase in metrics seen at some fractions may be compensated by higher dose to the tumor or better sparing of a separate OAR. In Figure 9, the changes in the three major criteria (bladder V90%, rectum V90%, and CTV D98%) are plotted to assess this interdependence. The larger plot includes all 240 fractions, while the in‐set plot excluded fractions where both the bladder V90% and rectum V90% were within our clinical threshold. For this small plot, if the value was within the clinical threshold for one of the metrics it was set to zero so that only differences above our threshold are highlighted. The same plot for V75% and V50% can be seen in FiguresS1 and S2.

In comparing the interdependence of the CTV, rectum, and bladder metrics, we found that changes in OAR metrics tended to be correlated. Thus, adaptation either improved the normal tissue sparing for both the bladder and rectum or both saw minor (0‐10%) dose increases. Almost all (92.5%) of the 240 fractions improved the CTV D98%. The average change in CTV D98% was 2.9% though improvements were on average slightly larger for cases where both the rectum and bladder saw more dose (4.6%) compared to those cases where toxicity in both rectum and bladder was reduced (0.6%). Out of 240 fractions, there was only one fraction where the V90% for both bladder and rectum were over the clinical thresholds (top right quadrant of in‐set plot). Adaptation for this fraction improved the dose to bladder by 13.9%, and rectum by 11.9%, while maintained CTV D98% the same (101%).

## 4 DISCUSSION

In this study of the Ethos adaptive platform, we investigated if an adaptive workflow using auto‐segmentation on daily CBCT images without expert edits could improve tumor coverage or normal tissue sparing for prostate patients. Eliminating the need for expert contour modifications at every fraction would improve the efficiency of the adaptive workflow and increase the potential for more widespread implementation. For instance, to adapt 20 of the 60 patients we currently treat daily on our Halcyon, we would need at least 5 extra minutes per patient for the Ethos software to auto‐segment, auto‐plan, and auto‐QA the plan. This would mean a total of 100 extra minutes of machine time per day. If we allowed another 5 minutes for segmentation edits, we could either adapt only 10 patients per day or extend the treatment day by an additional 100 min. Thus, minimizing the time required for adaptation while still ensuring safe treatments is key to maximizing the number of patients we can adapt. Additionally, because a CBCT driven adaptive approach cannot monitor intrafraction motion, it is critically important to minimize the time between image acquisition and the beginning of treatment. This workflow contrasts with another commercial adaptive solution that uses magnetic resonance image guided adaptive therapy (MRgRT) and can require up to 45 min per treatment fraction^22^ but has been largely directed at SBRT plans with reduced margin for error due to the smaller number of fractions and high dose per fraction.

Our results showed that adaptation with minor auto‐segmentation errors produced higher CTV doses for 92.5% of fractions and improved OAR sparing when organ metrics exceeded critical clinical thresholds without adaptation by 13.1% for the bladder V90% and 6.5% for the rectum V90%. However, a major auto‐segmentation error was also observed for one patient that if unnoticed and treated would have led to severe under‐dosage of his CTV since the adapted CTV_true_ D98% was equal to 42.7% ± 14.8%. This highlights the need for physician approval prior to the first adapted fraction. Thus, we anticipate a workflow where the physician reviews and edits the auto‐segmentation if necessary prior to the first treatment. Then, at subsequent fractions, the auto‐segmentation and plan are reviewed online by a trained physicist or therapist and approved offline by a physician to ensure the auto‐segmentation remains appropriate over time. This workflow mimics our current review process for IGRT treatments. Small errors in the auto‐segmentation noted by the physician at day 1 could be quickly corrected at each treatment. However, large errors, such as the one seen at every fraction for patient 25 in this study, would be too time consuming and thus those cases could be switched back to our current non‐adaptive standard of care workflow.

Rather than evaluating the auto‐segmentation with metrics such as the Hausdorff distance and dice similarity coefficient, we assessed the dosimetric impact on ground truth contours for the CTV, bladder, and rectum from plans created for the auto‐segmented CTV_auto_. Using the CTV_true_ D98% highlights the potential clinical impact and allows for a more intuitive assessment of whether the auto‐segmentation performed accurately enough for clinical use. We found that our current non‐adaptive clinical standard resulted in only 89% of fractions covering 98% of the CTV_true_ volume by the 95% isodose line. Daily adaptation was able to improve the frequency to 97% or 100% of treatment fractions respectively if the segmentation results were left as is or manually edited prior to re‐optimization.

When we examined inter‐patient variability, we also observed that four patients had larger improvements with adaptation than the rest of the cohort. Identifying characteristics of these patients prior to treatment or features from the anatomy‐of‐the‐day where these relatively larger improvements occur would help us recommend specific patients for daily adaptation and/or ration the total number of fractions at which we need to adapt thus further limiting the impact on the clinic while improving patient care. Identifying these characteristics will be the focus of a future study.

The auto‐segmentation of the OARs was highly accurate and only minimal changes were required to create Bladder_True_ and Rectum_True_. Importantly the changes made had minimal effect (<1%) on the DVHs, thus the DVH metrics reported by Ethos can be used to evaluate the safety of the adapted plan prior to its selection. In a few instances, we observed that the superior part of the bladder extended beyond the range of the CBCT image, and therefore could not be auto‐contoured completely resulting in an overall smaller volume. However, since the IOE tried to meet the clinical goals using this smaller bladder volume, the effect on the plan would be to overprotect the true bladder, and therefore the actual DVH metrics for bladder (V90,75,50) would be even lower than what were reported. When we compared the OAR metrics from the scheduled to the adapted plans, we found that adapting systematically lowered the OAR metric if it exceeded our clinical threshold with the scheduled plan. Thus, it is possible that daily adaptation could result in even lower toxicity rates for this population. In evaluating the interplay between the CTV and OAR metrics, we observed that all fractions where the adapted plan resulted in higher rectal or bladder dose were directly compensated with increased CTV_true_ D98%, except one case where rectum V90% was increased by 12.1% and CTV D98% was reduced by 0.7%. Thus, the adapted plans created by the Ethos auto‐plan software uses the prioritized goal list to reach similar compromises seen in manual planning.

Our study had a few limitations. Specifically, we analyzed 10 fractions for 25 patients instead of the full course of radiotherapy. Our choice of 10 fractions was driven by a few studies that showed that the amount of patient variability can be accurately assessed from the first five fractions and have even prospectively used the motion of the prostate measured over only the first week's daily imaging to successfully create patient‐specific PTV margins.^24^, ^25^ Thus, we believe our results that doubled the number of fractions per patient to assess intra‐patient variability are representative of the scale of motion that are likely to occur for these patients. Additionally, we observed that errors in the auto‐segmentation which were not attributable to motion were systematic. For example, for patient 25, the auto‐segmentation incorrectly contoured the CTV at each fraction, consistently truncating the superior section of the prostate where it was surrounded by bladder. However, because we have not yet performed a secondary analysis for the boost or prostate only plans, it is possible that the benefits we found for the CTV are slightly overestimated. This is because in current practice the boost CTV volume includes only the prostate. And while the prostate can shift position day‐to‐day, we can already correct for most of this motion with our IGRT‐based couch shifts and thus ensure full target coverage with prescription dose.

Another potential limitation is the fact that we did not accumulate the dose from the adapted or scheduled plans over the 10 fractions and scale it for a full course of radiotherapy. Accumulating the dose could demonstrate that decreases in the CTV D98% average out because they occur in slightly different positions in the CTV each day. In our analysis, we tracked the dose from each fraction independently to assess overall trends in the CTV or OAR metrics. Thus, our results may present a slight overestimate of the effects of daily adaptation. This approach is also common in adaptive studies,^26^ because it is more conservative and avoids compounding errors from an inaccurate dose deformation algorithm.

In this study, we used 12‐field IMRT to create the scheduled and adapted plans. Ethos also has the capability to calculate and deliver multi‐arc volumetric modulated arc therapy (VMAT) plans which are commonly used for prostate radiotherapy. However, VMAT plans take longer to optimize for each adapted fraction adding an unnecessary treatment delay and in a preliminary analysis did not result in improved dose distributions compared to the 12 field plans. Additionally, in a recent study by Sibolt et al, they also found that the Ethos generated IMRT plans resulted in superior dose metrics compared to both 2‐ and 3‐arc VMAT plans generated by Ethos and thus used IMRT plans for clinical adaptive treatments.^27^

Finally, while we evaluated a fully automated workflow in this study, we are aware that this implementation strategy will likely not be palatable for most clinics. Our results can serve as an extreme upper bound of what is possible with a self‐driving adaptive system and help to demonstrate that necessitating a physician's presence prior to each beam‐on is likely not necessary. Instead, a physician can be required at day one to evaluate if the CTV auto‐segmentation is reasonable and what systematic edits should be performed at subsequent fractions. Then a trained non‐MD staff member (e.g., a medical physicist) can perform an initial review with minor edits of the CTV auto‐segmentation at subsequent treatments with offline review and evaluation by physicians. In our experience and a recent study,^27^ these edits require only 1‐3 min for prostate cancer and could be completed immediately by a trained person who remains at the machine for the entirety of all adaptive treatments. This maintains clinical efficiency and decreases the number of urgent interruptions to physicians during their day. Alternatively, daily manual edits by physicians could be performed for patients who have shortened courses of treatment such as prostate SBRT regimens where 36.25 Gy is delivered in five fractions. In these cases, the daily dose is higher, and thus even minor segmentation errors are more consequential. Lastly, while we evaluated the potential for improvement in prostate cancer the same approach may see more impactful gains in treatment sites where off‐line adaptation is already frequently required such as head & neck and gynecological cancers.

## 5 CONCLUSION

We have evaluated the Ethos adaptive workflow using a retrospective cohort of 25 prostate cancer patients and no manual edits to the auto‐segmentation. We confirmed that for 24 of the patients, the auto‐segmentation results from Ethos were accurate enough without manual edits to improve CTV D98% and reduce normal tissue dose for structures that would otherwise exceed clinical thresholds. However, for one case where the prostate overlapped with the bladder, the auto‐segmentation substantially under‐contoured the prostate at every fraction leading to adapted plans that would have underdosed the CTV. Thus, a careful review of the auto‐segmentation is required prior to delivery of the first adapted fraction while a systematic offline review of subsequent fractions can be used to maintain efficiency. This approach would reduce the amount of time required to adapt at treatment, reduce the risk of intra‐fraction motion, and increase the number of patients that can be adapted in a standard clinical treatment day.

## AUTHOR CONTRIBUTIONS

MM collected and analyzed the data and wrote the manuscript. BR evaluated/edited the CTV contours and revised the manuscript. KK aided in project design, consulted on all project aspects, and revised the manuscript. KLM aided in project design, contributed to data analysis and interpretation, and revised the manuscript. XR designed the study, aided in data collection, oversaw data analysis and interpretation, and helped write the manuscript. All authors approved the final manuscript version.

## CONFLICT OF INTEREST

X.R. had a lab services agreement with Varian Medical Systems. K.L.M. reports income for personal consulting and speaker's honoraria from Varian Medical Systems.

## Supporting information

Supporting information
