# Supplementary material for: Prospects for daily online adaptive radiotherapy via ethos for prostate cancer patients without nodal involvement using unedited CBCT auto‐segmentation
Source: J Appl Clin Med Phys. 2021 Aug 25;22(10):82–93. doi: 10.1002/acm2.13399 (PMC8504605; doi:10.1002/acm2.13399)
Supplement: Supplementary file 1 — Supporting information [file ACM2-22-82-s001.docx]

**Title:**

Prospects for Daily Online Adaptive Radiotherapy via Ethos for Prostate Cancer Patients without Nodal Involvement Using Unedited CBCT Auto-Segmentation

**Authors:**

Mojtaba Moazzezi^1^, Brent Rose^1^, Kelly Kisling^1^, Kevin L. Moore^1^, and Xenia Ray^1^

**Affiliations:**

^1^Department of Radiation Medicine and Applied Sciences, University of California San Diego, La Jolla, California, 92093, USA

**Corresponding Author:**

Xenia Ray

Radiation Oncology PET/CT Center

3960 Health Sciences Dr. MC 0865

La Jolla, CA, 92093

[xray@health.ucsd.edu](mailto:xray@health.ucsd.edu)

**Suggested Running Title:**

Online Adaptive Radiotherapy via Ethos for Prostate Cancer

**Author Contribution Statement:**

MM collected and analyzed the data and wrote the manuscript. BR evaluated/edited the CTV contours and revised the manuscript. KK aided in project design, consulted on all project aspects, and revised the manuscript. KLM aided in project design, contributed to data analysis and interpretation, and revised the manuscript. XR designed the study, aided in data collection, oversaw data analysis and interpretation, and helped write the manuscript. All authors approved the final manuscript version.

**Acknowledgments**

This work was supported in part by the Agency for Healthcare Research and Quality (AHRQ R01HS025440) and an internal seed grant from the Center for Precision Radiation Medicine.

**Conflict of Interest**

X.R. had a lab services agreement with Varian Medical Systems. K.L.M. reports income for personal consulting and speaker’s honoraria from Varian Medical Systems.
